# Cryopreserved vascular allografts for venous lengthening after robot-assisted living donor nephrectomy: a single institution experience

**DOI:** 10.3389/fsurg.2026.1816916

**Published:** 2026-04-14

**Authors:** Vincenzo Li Marzi, Gianluigi Adani, Alessio Pecoraro, Claudia Lucia Catucci, Giorgio Micheletti, Riccardo Campi, Nicoletta Mancianti, Giulio Bagnacci, Francesco Sessa, Guido Garosi, Sergio Serni

**Affiliations:** 1 Urology Unit, Department of Medicine Surgery and Neuroscience, University Hospital of Siena, Siena, Italy; 2 Kidney Transplant Unit, Department of Medicine Surgery and Neuroscience, University Hospital of Siena, Siena, Italy; 3 Unit of Urology and Renal Transplantation, Oncology Department, Careggi University Hospital, Florence, Italy; 4 Department of Experimental and Clinical Medicine, University of Florence, Florence, Italy; 5 Nephrology, Dialysis and Transplantation Unit, Department of Medical Science, University Hospital of Siena, Siena, Italy; 6 Unit of Diagnostic Imaging, Department of Medical, Surgical and Neuro Sciences, University of Siena, Siena, Italy

**Keywords:** allograft, anastomoses, healthcare, kidney transplantation, robotics

## Abstract

**Introduction:**

Living-donor kidney transplantation (LDKT) is the gold standard for end-stage renal disease. Traditionally, the left kidney is preferred for its longer vein. However, the “donor safety first” principle, combined with the transition to laparoscopic and robotic donor nephrectomy, has increased the frequency of using right-sided grafts or encountering “iatrogenically” shortened veins due to mechanical stapling. In this study, we report our preliminary experience evaluating the efficacy of cryopreserved vascular grafts for renal vein lengthening in LDKT to overcome anatomical vascular length limitations.

**Methods:**

All LDKT in this series were performed using a robotic-assisted laparoscopic approach. All procedures were carried out by a dedicated and experienced surgical team thanks to a cross-institutional partnership involving two regional University Hospitals. When necessary, cryopreserved venous allografts were employed to ensure adequate renal vein length. All transplants were carried out using a standard retroperitoneal approach in the iliac fossa.

**Results:**

From June 2024 to October 2025, nine living-donor kidney transplants were performed. The donor cohort included 7 females and 2 males with a median age of 58 years (IQR 51–69), while the recipient cohort included 4 females and 5 males with a median age of 39 years (IQR 23–55). Cryopreserved venous allografts were used in 5/9 LDKT (55.5%), following right kidney procurement. Cold ischemia time was higher in grafts requiring vascular extension than in those without elongation (median 139 min [IQR 130–141] vs. 115 min [IQR 107–121], respectively; *p* < 0.05). Rewarming time was also longer in the vessel extension group (median 38 min [IQR 37–40] vs. 33.5 min [IQR 31–35], respectively; *p* = 0.6). No intraoperative or high-grade postoperative complications were observed. At a median follow-up of 10 months (IQR 8–17), there were no deaths or graft losses. The median serum creatinine level at last follow-up was 1.6 mg/dL (IQR 1.2–1.7).

**Conclusion:**

Renal vein lengthening with cryopreserved vascular grafts is a valuable tool in modern transplantation, addressing short veins—common in right-sided grafts and after laparoscopic or robotic stapling—and complex recipient venous anatomy. By enabling safer anastomoses, this technique supports excellent graft function while preserving donor safety.

## 1 Introduction

Living-donor kidney transplantation (LDKT) represents the gold standard treatment for end-stage renal disease (ESRD), as it provides superior graft survival and health-related quality of life compared to deceased donor transplantation (1, 2). Traditionally, the left kidney has been the preferred choice for retrieval due to its longer renal vein, which provides a more favorable vascular pedicle for anastomosis in the recipient. However, the surgical paradigm, guided by the “donor safety first” principle, frequently requires the use of the right kidney, which is anatomically characterized by a shorter vein.

In the current era, the challenge of a short renal vein is no longer confined to right-sided grafts. In fact, as well highlighted by Barandiaran Cornejo et al., the evolution from Carrel's pioneering techniques to the widespread adoption of laparoscopic and robot-assisted donor nephrectomy has introduced new technical issues (3). The use of mechanical staplers for vascular sectioning, while enhancing donor safety, often results in a significant reduction of the available vein length for the recipient's anastomosis. This “iatrogenic” shortening, together with the increasing age and burden of comorbidities among both donors and recipients, and the imperative to expand the donor pool, creates a surgical scenario where open kidney transplantation may be particularly challenging, especially in obese patients (2, 4).

To address these anatomical and technical limitations, various vascular reconstruction strategies have been proposed, ranging from autologous vein transpositions (5) to the less common use of synthetic materials (6). Among these, cryopreserved vascular grafts (biological allografts) allow for a standardized reconstruction with optimal vascular geometry, avoiding the additional morbidity associated with autologous vessel harvesting (7).

In this study, we report our preliminary experience with the use of cryopreserved vascular grafts for renal vein lengthening in LDKT. Our aim is to evaluate how this biological interposition can overcome the surgical challenges that can arise in this setting, ensuring recipient success without compromising donor safety or minimally invasive principles.

## 2 Materials and methods

All living donor nephrectomies (LDN) in this series were performed using a robotic-assisted laparoscopic approach (RALDN). The surgical protocol follows the well-codified robotic program which led to the standardization of the procedure (8). All procedures were performed by a dedicated surgical team, led by a seasoned robotic and transplant surgeon (S.S.), through a cross-institutional partnership between two regional University Hospitals (Siena University Hospital and Careggi University Hospital). In our series, right kidney procurement was indicated by complex left-sided vascular anatomy in all cases. Additionally, the right graft was preferred whenever preoperative renal scintigraphy revealed a functional disparity of ≥10% favoring the left side. Vascular control of both the renal artery and vein during nephrectomy was achieved using a surgical stapler (ECHELON FLEX™ Powered Vascular Stapler). All grafts were maintained in a sterile, ice-cold preservation solution until implantation. Each kidney was gravity-perfused with Celsior solution until the effluent was clear, using a minimum volume of 1 L per graft. When necessary, cryopreserved venous allografts were employed to ensure adequate renal vein length. These allografts were retrieved from both donors after brain death (DBD) and donors after circulatory death (DCD), and were processed and provided by the tissue bank of the Department of Immunohematology and Transfusion Medicine at Policlinico S. Orsola-Malpighi (Bologna, Italy).

Recipient surgeries and bench-table preparation were carried out by two experienced transplant surgeons (V.L. and G.A.) using a standard retroperitoneal approach in the iliac fossa. In the entire cohort, end-to-side anastomoses to the external iliac vessels (vein and artery) were systematically performed. For the venous anastomoses and the reconstruction of the allograft to the renal vein, 5-0 or 6-0 polypropylene sutures were used, while 6-0 polypropylene was employed for the arterial anastomoses. A duplex ultrasound-based follow-up protocol was implemented for the early detection of severe postoperative vascular complications according to established protocol (9).

Cold ischemia time (CIT) was defined as the time of cold storage between kidney harvesting and kidney transplantation, while re-warming time as the time between the beginning of vascular anastomosis and its reperfusion (10). Delayed graft function (DGF) was defined as the need for dialysis in the first postoperative week (10). Postoperative complications were reported according to the Clavien-Dindo grading system (11). High-grade postoperative complications (HGCs) were defined as Clavien-Dindo grade >2. Donors’ assessment, postoperative management of recipients, and follow-up after kidney transplantation were performed according to established Guidelines (10).

## 3 Results

From June 2024 to October 2025, nine LDKTs were performed (Table 1). The donor cohort included 7 females and 2 males with a median age of 58 years (IQR 51–69), while the recipient cohort included 4 females and 5 males with a median age of 39 years (IQR 23–55).

**Table 1:** Pre- and intraoperative characteristics of recipients and grafts. In all recipients, both venous and arterial anastomoses were performed in an end-to-side fashion onto the external iliac vein and the external iliac artery.

Case	Recipient Age (yrs)/Sex	Etiology of ESRD	Dialysis (mos)	Donor Age (yrs)/Sex/Relation	Previous Major Surgery	Date of KT	Donated kidney/Iliac Fossa	Post-KT Follow up mos	CVA	Vascular Graft Pedicle/Anastomosis Suture Techniques	CIT (min)	Re-Warming time (min)
1	49/F	ADPKD	Hemodialysis (2)	51/F/Sister	No	06-27-2024	Right/Right	20	inferior vena cava	• 2 veins: creation of a venous common trunk and extension using inferior vena cava allograft (anastomosed with running sutures) • 2 arteries: reconstruction of dual arteries into a common arterial trunk anastomosed with interrupted sutures	130	38
2	40/M	Unknown	Peritoneal Dialysis before 1st KT (34) Hemodialysis before 2nd KT (24)	69/F/Blood-related Aunt	KT in 2014	07-25-2024	Right/Left	19	inferior vena cava	• Single vein: extension using inferior vena cava allograft (anastomosed with running sutures) • Single artery anastomosed with interrupted sutures	141	37
3	35/F	Multifactorial	Hemodialysis (20)	63/F/Mother	Pediatric pelvic sarcoma resection	09-12-2024	Right/Left	17	inferior vena cava	• Single vein: extension using inferior vena cava allograft (anastomosed with running sutures) • Single artery anastomosed with interrupted sutures	142	40
4	28/F	IgA Nephropathy	Preempitive	67/M/Father	No	10-03-2024	Right/Right	16	inferior vena cava	• Single vein: extension using inferior vena cava allograft (anastomosed with running sutures) • Single artery anastomosed with interrupted sutures	139	35
5	23/F	Left Renal Agenesis and Right Renal Hypoplasia	Preempitive	58/M/Father	No	04-10-2025	Left/Right	10	No	• Single vein anastomosed with running sutures • Single artery anastomosed with interrupted sutures	125	29
6	57/M	Alport Syndrome	Peritoneal Dialysis (21) Hemodialysis (4)	55/F/Wife	No	05-08-2025	Right/Right	9	No	• Single vein anastomosed with running sutures • Single artery anastomosed with interrupted sutures	110	35
7	31/M	Multifactorial	Preempitive	61/F/Mother	Heart transplantation in 2022	06-12-2025	Right/Right	8	inferior vena cava	• Single vein: extension using inferior vena cava allograft (anastomosed with running sutures) • Single artery anastomosed with interrupted sutures	120	42
8	55/M	ADPKD	Preempitive	54/F/Wife	No	07-10-2025	Left/Right	7	No	• Single vein anastomosed with running sutures • Single artery anastomosed with interrupted sutures	100	32
9	39/M	IgA Nephropathy	Hemodialysis (6)	58/F/Mother	No	10-02-2025	Left/Right	4	No	• Single vein anastomosed with running sutures • Single artery anastomosed with interrupted sutures	120	35

ESRD, End-Stage Renal Disease; ADPKD, Autosomal Dominant Polycystic Kidney Disease; CVA, Cryopreserved Vascular Allografts; CIT, Cold Ischemia Time; Re-warming time, time between the beginning of vascular anastomosis and graft reperfusion.

**Figure 1:**
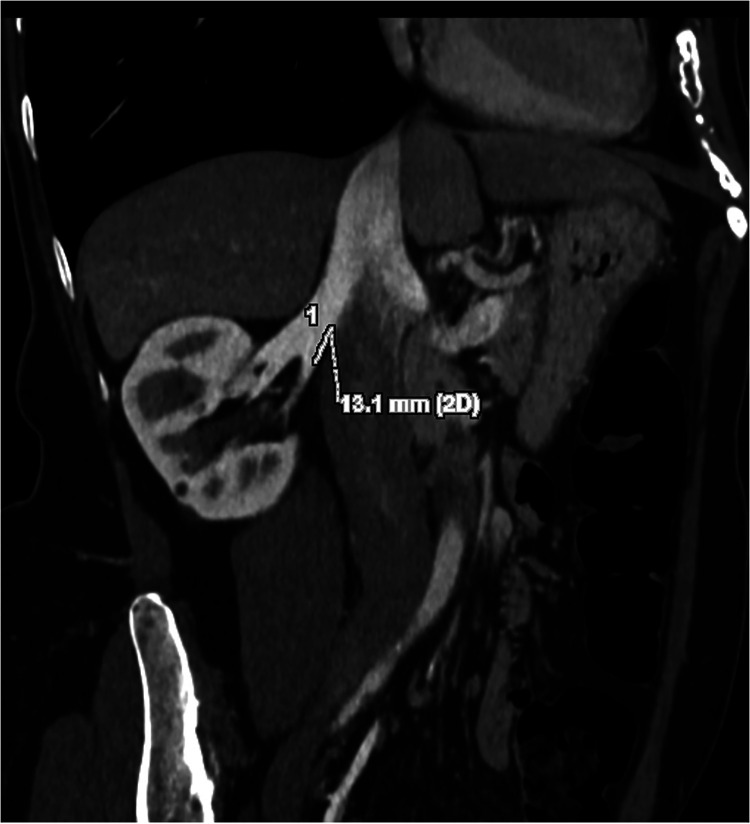
Preoperative donor CT scan (multiplanar coronal-oblique reconstruction related to case #7 in Table 1). The right renal vein measures 13 mm from the caval ostium to the hilar division.

**Figure 2:**
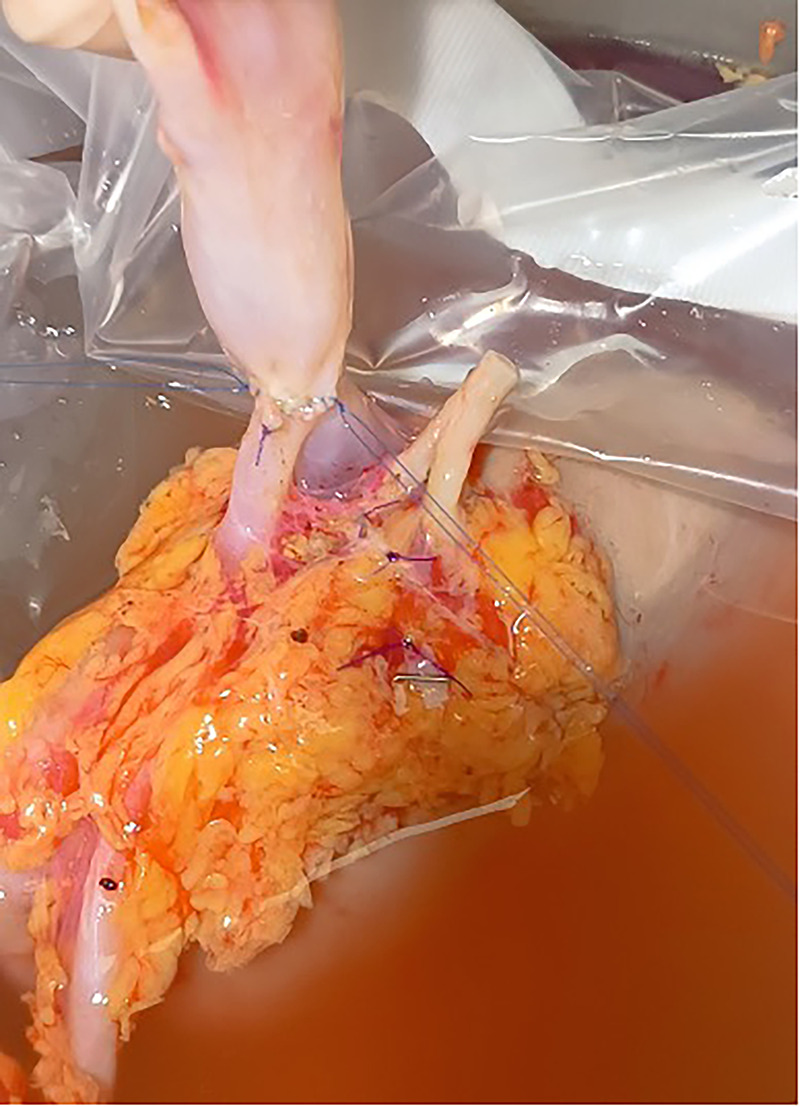
Right renal vein elongation during back-table surgery (case #7). A cryopreserved inferior vena cava allograft is anastomosed to the renal vein using double running 5-0 polypropylene sutures.

**Figure 3:**
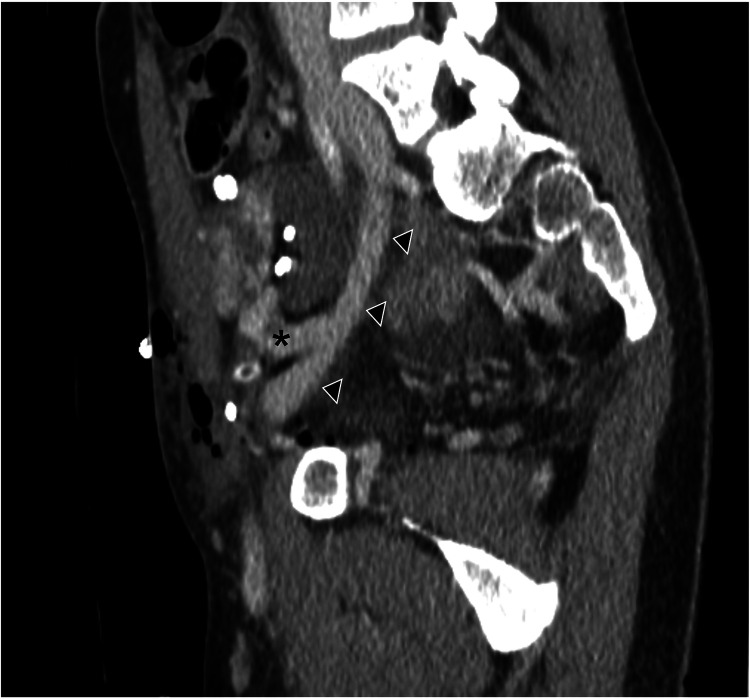
Postoperative recipient CT scan (multiplanar sagittal-oblique reconstruction related to case #7 in Table 1). Arrowheads indicate the course of the right external iliac vein; the asterisk indicates the cryopreserved vena cava patch. On postoperative day 2, the recipient underwent a CT scan for hypotension.

Cryopreserved venous allografts (Figures 1–3) were utilized in five recipients (5/9, 55.5%). In all five cases, the right kidney was transplanted. Specifically, the surgical complexity was further increased in two cases: one graft presented with dual renal arteries and veins, while another involved a recipient undergoing transplantation in the left iliac fossa who had a surgical history of pelvic sarcoma resection during childhood.

The median CIT was 24 min longer in grafts requiring vascular extension compared to those without elongation (139 min [IQR130-141] vs. 115[IQR 107-121] respectively; *p* < 0.05). Furthermore, the re-warming time was marginally prolonged in the vessel extension group compared to the other recipients (38 min [IQR 37-40] vs. 33.5 min [IQR 31-35], respectively; *p* = 0.6). No intraoperative or high-grade postoperative complications were observed among recipients or donors. The median hospital stay for donors was 6 (6–19) days. In two cases, persistent lymphorrhea necessitated delayed drain removal at 13 and 19 days. Regarding the recipients, the median hospital stay was 16 (15–35) days for the venous lengthening group and 15 (13–22) days for the group without lengthening. A urinary tract infection occurred in recipient #2 (Clavien-Dindo grade 2), requiring prolonged antibiotic therapy. Recipient #7, who had a history of prior heart transplantation, experienced hypotension related to acute rejection and to localized hematoma, which required adjustment of the immunosuppressive therapy and blood transfusions (Clavien-Dindo grade 2).

At a median follow-up of 10 months (IQR 8-17), no deaths or graft losses were recorded. The median creatinine level at the last follow-up was 1.6 mg/dL (IQR 1.2-1.7) (Table 2). Duplex ultrasound-based follow-up revealed no stenosis or other vascular abnormalities.

**Table 2:** Longitudinal serum creatinine (sCr) levels (mg/dL) and clinical follow-up findings for the kidney transplant recipients at 1 and 3 months, and last available follow-up.

Case	sCr 1st mounth	sCr 3rd mos	sCr at last Follow-up (date)	Clinical Follow-up Findings
1	1.4	1.7	0,9 (Feb 2026)	-
2	2.5	1.8	1.7 (Feb 2026)	-
3	2.3	3.8	1.6 (Feb 2026)	Successful heart transplantation 1 year after KT
4	1.1	1.1	1.0 (Jan 2026)	-
5	1.6	1.6	2.0 (Feb 2026)	Ureteral stenosis successfully treated with antegrade balloon dilation and Double-J stenting at 3 months post-KT; the stent was subsequently removed after one month
6	1.1	1.3	1.2 (Jan 2026)	-
7	4.8	2.8	3.0 (Dec 2025)	-
8	1.3	1.5	1.5 (Jan 2026)	-
9	2.0	1.5	1.6 (Feb 2026)	-

## 4 Discussion

The historical preference for the left kidney in living-donor kidney transplantation (LDKT) is primarily due to the longer left renal vein. In our practice, right kidney selection is driven by anatomical complexities, such as multiple vessels, a history of previous surgery such as left-sided surgery (also endoscopic) for urolithiasis, or a functional disparity of ≥10% favoring the left side. This approach is consistent with our fundamental policy of harvesting the “worst” kidney to prioritize and ensure long-term donor safety. Yet, as well highlighted by Barandiaran Cornejo et al. (3), renal transplantation is a continuously evolving field—progressing from the pioneering vascular suture techniques of Alexis Carrel to the current era of robotics and artificial intelligence. Within this evolving landscape, cryopreserved vascular grafts may serve as a bridge, combining vascular principles with cutting-edge technological advances.

In this context, our study offers meaningful insights for both clinical practice and future research.

First, to our knowledge, this is the first series assessing the safety and feasibility of using cryopreserved vascular allografts for venous lengthening following RLDN. While the left renal vein is anatomically longer, the adoption of laparoscopic and robotic approaches has modified surgical practice, increasing the number of right-sided kidney harvested.

The use of mechanical staplers for vascular section can reduce the available vessel length, as a safety margin on the donor side is required, removing precious millimeters from the graft's pedicle. Recipient-side anatomy further complicates transplantation. Increasing donor and recipient age correlates with higher prevalence of comorbidities. Advanced atherosclerosis, obesity, prior deep vein thrombosis, or inadequate bilateral iliac veins can render standard implantation sites unviable. In such scenarios, successful graft outflow has been achieved by anastomosing the renal vein to alternative targets, including the portal, mesenteric, or gonadal veins (12–14). Cryopreserved vascular grafts can serve safely as interposition conduits, enabling tension-free, hemodynamically stable reconstructions even in complex vascular cases.

Second, despite the limited sample size, the observed increases in CIT and rewarming time were not clinically significant and did not negatively impact outcomes.

In the five cases, venous elongation facilitated a smoother procedure, mitigating challenges associated with short renal veins. Favorable outcomes—including absence of vascular complications, stable renal function, and positive postoperative Doppler ultrasound findings—demonstrate that venous allograft lengthening is both feasible and safe in our preliminary experience.

Extending renal vessels using banked tissues allows optimal management of anatomical variations, such as multiple renal veins or “iatrogenic” shortening, as discussed by Fallani et al. (7). Unlike autologous techniques - such as gonadal vein interposition (13), iliac vessel transposition (5), or vein cuff interposition (14) - cryopreserved allografts provide a robust, high-caliber conduit without requiring additional surgical sites. Puche-Sanz et al. demonstrated that cryopreserved iliac artery allografts effectively extend the right renal vein with excellent long-term patency (15, 16).

Biological tissues remain the gold standard in renal transplantation, whereas synthetic vascular prostheses are rarely used due to concerns about long-term patency, infection risk under immunosuppression, and late thrombosis. Tinay et al. described encouraging long-term results with Dacron grafts (6), yet this approach continues to be a niche solution rather than standard practice. In cases of short renal veins or challenging recipient pelvic anatomy, biological allografts offer superior integration and safety, allowing surgeons to prioritize donor safety and minimally invasive approaches without compromising recipient outcomes. Although back-table reconstruction slightly increases CIT, it prevents intraoperative complications, such as kinking or tearing, which are far more detrimental to graft survival (17, 18).

Finally, our study demonstrates that open kidney transplantation can be safely performed after RLDN, even when harvesting a right-sided graft. In this context, the use of cryopreserved vascular allografts for venous lengthening may support surgeons to overcome the technical challenges of short renal vessels, especially in cases where robot-assisted kidney transplantation is not feasible and/or available (19). Indeed, in our current clinical setting, the decision to perform renal vein elongation has also been influenced by the limited availability of robotic platforms. With only one system accessible at our center, we prioritize its use for the donor nephrectomy rather than the recipient implantation. We believe that privileging donor safety through a robot-assisted approach is of paramount importance, as it ensures a minimally invasive procedure with significantly reduced surgical impact and enhanced recovery.

In the near future, the expansion of robotic programs and the availability of more than one surgical platform within a single center could reduce the need for renal vein elongation. As highlighted in recent literature, the superior visualization and dexterity offered by robotic systems provide significant safety advantages when performing venous anastomoses compared to conventional open procedures, in both deceased and living donor transplantation (20, 21). Beyond these benefits, robotic surgery facilitates the integration of advanced technological adjuncts, such as intraoperative indocyanine green fluorescence vascular imaging and three-dimensional augmented reality reconstructions for precise anatomical navigation (22, 23).

Despite its novelty, our study has several limitations. First, this is a prospective series with a small sample size. Second, the decision to use cryopreserved vascular allografts for venous lengthening was made by the transplant team on a case-by-case basis, introducing potential selection bias. Third, both RLDN and open kidney transplantation were performed by highly experienced surgeons, which may limit the generalizability of our findings. Finally, the follow-up period was relatively short, restricting long-term outcome assessment.

In conclusion, renal vein lengthening using cryopreserved vascular grafts represents a valuable and versatile tool in modern transplantation. It effectively addresses the challenge of short veins - common in right-sided grafts or following laparoscopic and robotic stapling - and provides a reliable solution for complex recipient venous anatomy. To our knowledge, this is the first study reporting the use of cryopreserved vascular allografts specifically in robot-assisted LDKT. By converting potentially high-risk anastomoses into technically safe reconstructions, this approach ensures excellent functional outcomes for recipients while maintaining the highest standards of safety for living donors.

## Data Availability

The raw data supporting the conclusions of this article will be made available by the authors, without undue reservation.
